# Integration of Microfluidics, Photonic Integrated Circuits and Data Acquisition and Analysis Methods in a Single Platform for the Detection of Swine Viral Diseases

**DOI:** 10.3390/ani11113193

**Published:** 2021-11-09

**Authors:** Georgios Manessis, Christos Mourouzis, Amadeu Griol, David Zurita-Herranz, Sergio Peransi, Carlos Sanchez, Alessandro Giusti, Athanasios I. Gelasakis, Ioannis Bossis

**Affiliations:** 1 Laboratory of Anatomy and Physiology of Farm Animals, Department of Animal Science, Agricultural University of Athens (AUA), Iera Odos 75 Str., 11855 Athens, Greece; gmanesis@aua.gr (G.M.); gelasakis@aua.gr (A.I.G.); 2 CyRIC, Cyprus Research and Innovation Centre Ltd., 28th Octovriou Ave 72, Off. 301, Engomi, Nicosia 2414, Cyprus; c.mourouzis@cyric.eu (C.M.); alessandro@cyric.eu (A.G.); 3 Nanophotonics Technology Center, Universitat Politècnica de València, Camino de Vera s/n Building 8F, 46022 Valencia, Spain; agriol@upvnet.upv.es (A.G.); dazuher@ntc.upv.es (D.Z.-H.); 4 Lumensia Sensors S.L., Camino de Vera, s/n, K-Access, Building 8F 3th-Floor, 46022 Valencia, Spain; speransi@lumensia.com (S.P.); csanchez@lumensia.com (C.S.); 5 Laboratory of Animal Husbandry, Department of Animal Production, School of Agriculture, Faculty of Agriculture, Forestry and Natural Environment, Aristotle University of Thessaloniki, 54124 Thessaloniki, Greece

**Keywords:** Point of Care (POC), diagnostics, Photonic Integrated Circuits (PICs), microfluidics, Porcine Parvovirus (PPV), Porcine Circovirus 2 (PCV-2), oral fluids, validation

## Abstract

**Simple Summary:**

The control of several swine viral diseases relies mainly on evidence-based prevention protocols due to the lack of effective treatments or vaccines. To design these protocols, laboratory investigation of viral infections is critical to confirm their occurrence and determine their epizootiology. However, laboratory confirmation of certain swine viral diseases is a time-consuming and labor-intensive process, requiring scientific personnel with relevant expertise. Point-of-Care (POC) diagnostics are tests and devices that provide clinically relevant information on-site, facilitating decision-makers to swiftly take countermeasures for disease control. In the present study, novel photonic biosensors were integrated into a single, automated POC device that can record and analyze changes in the sensors’ refractive index, allowing the detection of Porcine Parvovirus (PPV) and Porcine Circovirus 2 (PCV-2) in oral fluids within 75 min. The objective of this work was to validate this device using reference and field samples (oral fluids). The system was able to detect PPV and PCV-2 in oral fluid samples satisfactorily. The device can be directly deployed in farms for the fast diagnosis of these diseases, contributing to farm biosecurity.

**Abstract:**

Viral diseases challenge the health and welfare of pigs and undermine the sustainability of swine farms. Their efficient control requires early and reliable diagnosis, highlighting the importance of Point of Care (POC) diagnostics in veterinary practice. The objective of this study was to validate a novel POC system that utilizes Photonic Integrated Circuits (PICs) and microfluidics to detect swine viral pathogens using oral fluids and Porcine Parvovirus (PPV) and Porcine Circovirus 2 (PCV-2) as proofs of concept. The sensitivity and specificity of the device were calculated for both viruses, and Receiver Operating Characteristic (ROC) curves were drawn. PPV had an Area Under Curve (AUC) value of 0.820 (95% CI: 0.760 to 0.880, *p* < 0.0001), and its optimal efficiency threshold of detection shifts was equal to 4.5 pm (68.6% sensitivity, 77.1% specificity and Limit of Detection (LOD) value 10^6^ viral copies/mL). PCV-2 had an AUC value of 0.742 (95% CI: 0.670 to 0.815, *p* < 0.0001) and an optimal efficiency threshold of shifts equal to 6.5 pm (69.5% sensitivity, 70.3% specificity and LOD 3.3 × 10^5^ copies/mL). In this work, it was proven that PICs can be exploited for the detection of swine viral diseases. The novel device can be directly deployed on farms as a POC diagnostics tool.

## 1. Introduction

Pig production accounts for approximately 35% of total meat production [1,2]. Increased demand for pork meat and products thereof has driven the use of intensive farming management systems and higher stocking densities to reduce production costs. Increased stocking density has led, in turn, to a higher risk of pathogen transmission [3]. At the same time, the expansion of livestock trade networks and inefficient surveillance programs have accelerated the emergence of transboundary infectious diseases [4,5]. Among these, viral diseases are of utmost importance for pig production due to (i) their transmission dynamics, (ii) the lack of efficient treatment, and (iii) the limited available preventive measures (e.g., hygiene and biosecurity measures, vaccines) to mitigate their spread in pig populations.

Two viral swine diseases of major significance are porcine parvovirus (PPV) and porcine circovirus 2 (PCV-2). PPV is a main cause of reproductive failure in swine, and its clinical manifestation is described by the acronym SMEDI (stillbirth, mummification, embryonic death, and infertility) [6]. PCV-2 is associated with post-weaning multisystemic wasting syndrome (PMWS) of swine [7]. PMWS is characterized by progressive weight loss, respiratory signs, and low morbidity jaundice. Nonetheless, PMWS can cause high mortality rates in 5–12-week-old pigs [8]. Both of these viruses may not have high fatality rates, but they affect productivity and have significant economic impacts, especially in low-input farming systems [9,10], making reliable Point-of-Care (POC) testing a priority for cost-effective disease control and management.

Currently, the period between clinical observation and laboratory confirmation for most swine viral diseases, including PPV and PCV-2, varies from days to weeks depending on the diagnostic assay used, and centralized laboratories and specialized personnel are frequently required [11]. Conventional methods for pathogen detection include some form of PCR, immunoassays, and cell cultures, all of which are labor-intensive and time-consuming [12]. In any case, early diagnosis of swine viral infections is necessary to control their spread, minimize their impact on production, and limit socioeconomic consequences that could affect the sector’s sustainability. Subsequently, the need for rapid and reliable tools with the potential to be used on-field has emerged.

POC testing refers to diagnostic devices and tests that are exploited on-site to provide clinical information or rapid confirmation of health-challenging conditions and diseases [13]. Common POC tests are dipstick and strip tests and Lateral Flow Assays (LFAs) (e.g., pregnancy tests in humans). Although handy and relatively cheap, these tests suffer from low sensitivity (even as low as 16%) and require trained personnel to avoid operational errors [14]. To overcome these issues, emerging technologies, including micro- and nano- fabrication, information and communication systems, photonics, and microfluidics have been exploited to produce next-generation POC devices using the lab-on-a-chip (LOC) device concept [15,16]. This trend is reflected by the translation of diagnostic technologies, such as PCR, LAMP and ELISA, to LOC devices for the detection of swine pathogens [17,18,19].

In this context, a fully-integrated bench-top analyzer was developed for rapid and reliable on-field detection of major swine viral pathogens using the European Union’s H2020 SWINOSTICS (swine diseases field diagnostics toolbox) project framework (www.swinostics.eu accessed on 11 October 2021). This analyzer represents a state-of-the-art POC diagnostic system for the detection of Porcine Parvovirus (PPV), Porcine Circovirus 2 (PCV-2), Swine Influenza (SIV), Porcine Reproductive and Respiratory Syndrome Virus (PRRSV), Classical Swine Fever Virus (CSFV), and African Swine Fever Virus (ASFV).

This system utilizes microfluidics and Photonic Integrated Circuits (PICs) for the label-free detection of viral antigens in complex biological matrices, including oral fluids, fecal samples, and sera. For this purpose, PICs have been functionalized with polyclonal or monoclonal antibodies as molecular recognition elements (MREs) to capture viral antigens, as described in detail by Montagnese et al. [20]. PICs are a promising platform for sensitive and specific sensing that have been used in gas sensing, biomedical diagnostics, and biochemical detection [21,22,23].

The objectives of the study were to (i) present, for the first time, the fully-integrated prototype device, the analysis protocol, the signal acquisition and analysis methods, and the refractive index shift calculation algorithm and (ii) validate the novel device using oral fluids. The validation of the system was focused on three key performance characteristics: sensitivity and specificity, the Limit of Detection (LOD), and virus detection in clinical samples.

## 2. Materials and Methods

### 2.1. Samples

As a PPV reference sample, the vaccine strain NADL-2 was used. In brief, the PPV NADL-2 strain was propagated in swine testicular cells, which were cultured at 37 °C in a 5% CO_2_ atmosphere in Dulbecco’s Modified Eagle Medium with 10% heat-inactivated FBS, 100 U/mL penicillin, and 100 μg/mL streptomycin. PPV was collected in the supernatant. Finally, the number of viral copies per ml of supernatant was estimated by real-time PCR (72 h post-inoculation).

PCV-2 samples were provided by the University of Veterinary Medicine Budapest (UVMB, Budapest, Hungary). For their preparation, PCV-2 strain R15 was isolated from pig lung tissue in 2009 using swine testicular cells. The isolate was stored at −80 °C in Dulbecco’s modified medium (DMEM). The isolate was propagated on the same cell line in 25 cm^2^ flasks with 15 mL DMEM. The cell culture medium was supplemented with 10% inactivated FBS, 50 U/mL penicillin, and 0.05 mg/mL streptomycin. For resuspension of the cells, phosphate buffer saline (PBS) containing 0.5% trypsin was used. Before every passage, cells were washed with 5 mL PBS. Incubation was performed at 37 °C in a 5% CO_2_ atmosphere, and virus growth was assessed by real-time PCR.

Oral fluid (n = 75) and fecal (n = 80) samples were retrieved at the pen level from 6 swine farms located in Central and Southern Greece. Samples were transported to the laboratory at 4–6 °C and processed within 24 h. After a freeze–thaw cycle, oral fluids were centrifuged at 12,000× *g* for 10 min, and supernatant was stored at −80 °C. Feces were incubated for 30 min in 20% *w*/*v* sucrose in PBS solution at 1:3 ratio. Afterwards, they were centrifuged at 3000× *g* for 20 min, and supernatant was collected and stored at −80 °C. All samples were serially filtered with filters with 5, 0.8, and 0.2 μm pore sizes prior to testing with the SWINOSTICS prototype.

### 2.2. Conventional PCR Assays

Viral PPV and PCV-2 DNA was isolated using the PureLink™ Viral RNA/DNA Mini Kit (Invitrogen, Carlsbad, CA, USA). The DNA isolation protocol was performed according to the manufacturer’s instructions using a standard volume of 200 μL per sample. Nucleic acids from each sample were eluted in 20 μL of elution buffer and stored at −20 °C.

PPV DNA was detected with conventional PCR using 3 different primer sets located within the NS and NS1 gene regions (Table 1). PCV-2 DNA was also detected with conventional PCR using 3 different primer sets located within the capsid protein gene, rep gen, and ORF1 regions. The primer sets and products of PPV and PCV-2 amplification are presented in Table 1. All conventional PCR assays were performed in a total volume of 25 μL consisting of 22.5 μL PCR 1.1 × SuperMix (Invitrogen, Carlsbad, CA, USA), 0.5 μL of 10 μM forward primer solution, 0.5 μL of 10 μM reverse primer solution, and 1.5 μL of template DNA. Cycling conditions were optimized for each of the 6 primer sets (Table 2). PCR products were analyzed in 2% agarose gel and stained with ethidium bromide. A 100 bp ladder (Thermoscientific, Vilnius, Lithuania) was used to assess amplicon length.

### 2.3. Quantitative PCR (qPCR) Assays

Quantitative PCR for the detection of viral DNA isolated from clinical samples was performed in triplicate using SYBR Green chemistry and the primer sets PPV_Set_2 (NS1 gene) and PCV2_Set_1 (Cap gene) for PPV and PCV-2, respectively. The reactions were performed in a total volume of 20 μL, consisting of 10 μL 2× PowerUp™ SYBR™ Green Master Mix with 500 nm ROX (Applied Biosystems, Vilnius, Lithuania), 0.5 μL of 10 μM forward primer solution, 0.5 μL of 10 μM reverse primer solution, 1 μL of template DNA, and 8 μL H_2_O. Cycling conditions were as follows: initial activation of UDG for 2 min at 50 °C, activation of the Dual-Lock polymerase for 2 min at 95 °C, 40 cycles of denaturation at 95 °C for 15 s, and annealing and extension at 60 °C for 1 min. Data were collected with a 7500 Real Time PCR System and analyzed with 7500 software, v.2.0.6 (Applied Biosystems).

Quantification of the viral load was performed with a standard curve generated from known amounts of PPV and PCV2 DNA, ranging from 10^10^ to 10^3^ genomes per PCR reaction, in duplicate. PPV and PCV-2 concentrations are expressed as the viral copy number per ml of sample.

### 2.4. POC Device

**Figure 1:**
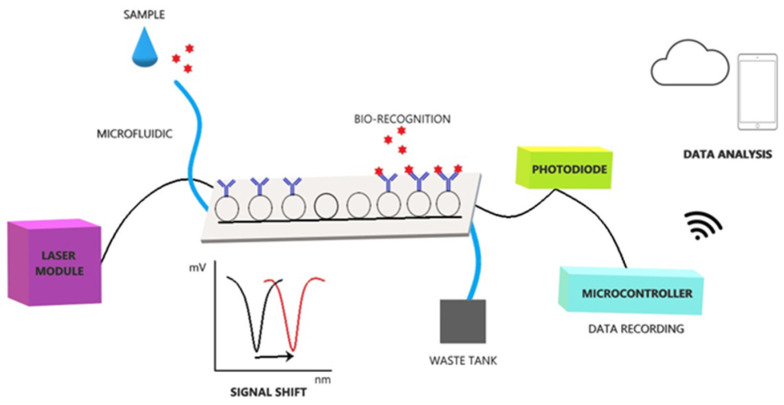
The novel POC system concept.

**Figure 2:**
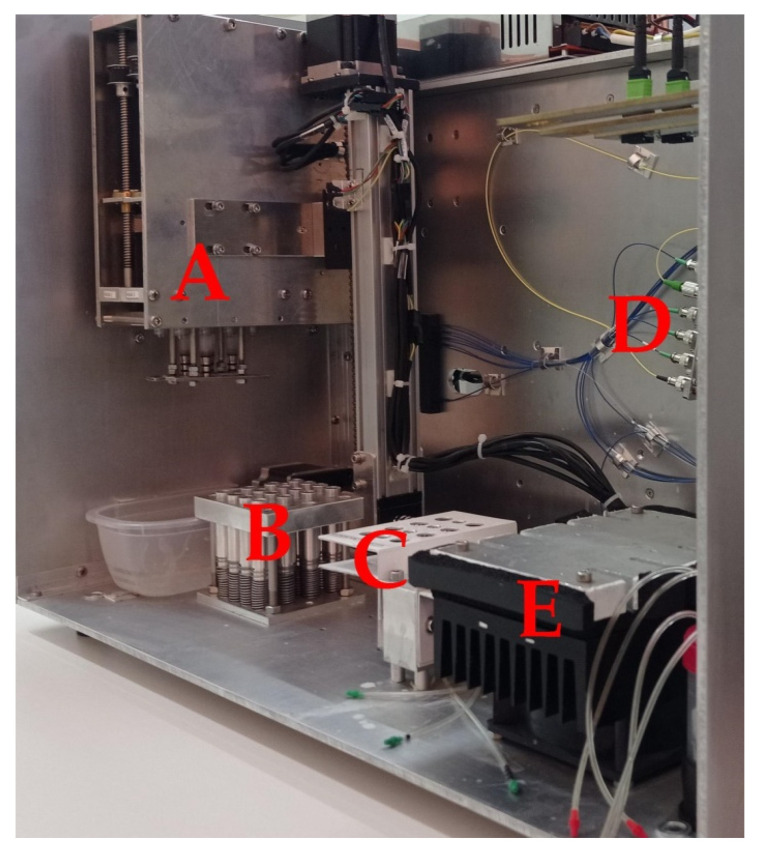
The novel POC system: (**A**) syringe system, (**B**) pipette tip holder, (**C**) buffer/sample holder, (**D**) optic fibers and optical splitter, (**E**) temperature control module.

The system follows a modular approach to efficiently integrate its components into a single, portable diagnostic platform with a size of about 40 × 50 × 60 cm (Figure 1). The system can be divided into three functional subsystems. The first one is the optics subsystem, which consists of (i) a tunable laser module (model PPCL200, Pure Photonics, Milpitas, CA, USA), operating at a wavelength range of 1549.3150 to 1550.9180 nm (the central point was 1550.1161 nm), (ii) optic fibers, and (iii) a fiber pigtailed InGaAs photodiode (75 μm, 9/125 SMF, FC/PC connector, 1 m, model PDINP0751FCA-0-0-01, Huntingdon, UK). The second subsystem refers to the mechanics/microfluidics subsystem which consists of (i) a syringe system that delivers the sample and the buffers to the sensors, (ii) motors that move the syringe system on the x- and z- axes, (iii) a microfluidics channel, (iv) a waste tank, and (v) a temperature control module (Figure 2) [20]. Finally, the firmware subsystem consists of (i) the microcontroller and its software, (ii) the arduino data logger, and (iii) an SD card. The microcontroller utilizes Bluetooth Low Energy technology to communicate with a tablet for the operation of the device. Experimental data are uploaded to a cloud platform via the tablet, generating real-time results. Features such as system operation, analysis progress, data collection/storage, and results are monitored via an android application in the tablet.

### 2.5. Sensors, Antibodies, and Biofunctionalization

At the core of the system lie its sensors. Photonic Integrated Circuits (PICs) utilize 8 ring resonators with immobilized antibodies on their surfaces. Following laser excitation at a continuous wavelength in the range of approximately 1.5 nm, each ring resonates at a specific wavelength, trapping that wavelength in the ring and preventing it from reaching the photodiode. This results in a measurable minimum in the wavelength spectrum, which can be detected by the photodiode. The capture of viral antigens via the antibodies results in a localized change in the refractive index which extends beyond the sensor’s surface [29]. This change modifies the resonant wavelength of the rings, causing a signal shift that is detected by the photodiode.

**Figure 3:**
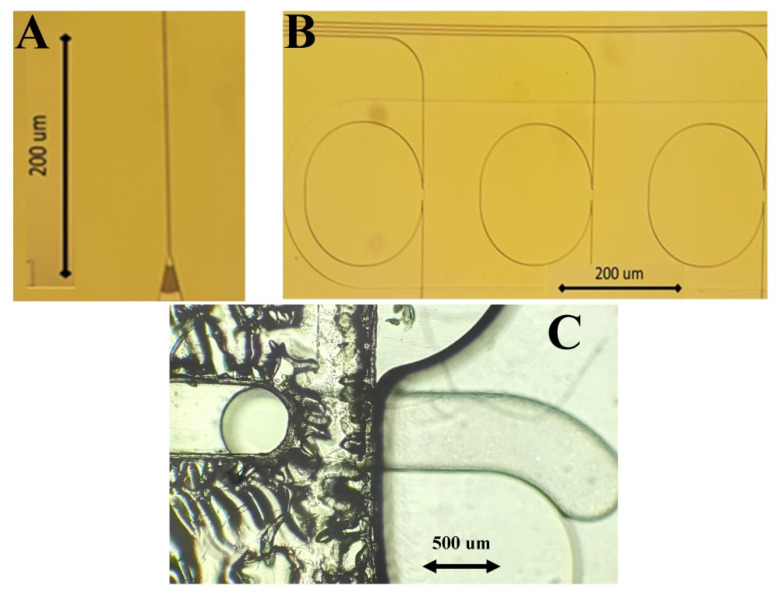
Microscopic images of PICs: (**A**) grating coupler, (**B**) ring resonators of PICs, and (**C**) buffer drop entering the PIC surface.

A total of 12 PICs were used for the study. PICs were provided by Universitat Politècnica de València Nanophotonics and Technology Center and Lumensia Sensors S.L. and were fabricated and functionalized as previously described by Griol et al. [30]. The dimensions of the microfluidic board of PICs were 7 × 3 cm, and the board was fabricated in cyclic olefin polymer (COP). The diameter of the microfluidic channels was 500 μm (Figure 3). Each PIC was functionalized with both anti-PPV and anti-PCV-2 antibodies to enable multiplex detection of these two viruses. Three out of the eight sensor rings (3/8) were functionalized with polyclonal anti-PPV antibodies (Rabbit Anti-Swine/Porcine Parvovirus VP2 antisera, Cat. No. PPVVP21-S, Alpha Diagnostic, San Antonio, TX, USA). Using the same concept, three out of the eight rings (3/8) were functionalized with polyclonal anti-PCV-2 antibodies (Rabbit Anti-Swine/Porcine Circovirus Type 2 Capsid Antibody, Cat. No. PA5-34969, Invitrogen, Carlsbad, CA, USA). The two remaining rings were functionalized with Bovine Serum Albumin (BSA) and acted as reference rings for the establishment of the signal baseline.

### 2.6. Analysis Protocol

The analysis protocol was optimized for the detection of PPV and PCV-2 in complex biological matrices, such as oral fluids and feces. The finalized analysis protocol included 5 consecutive steps:

1. The buffer step: The buffer used was PBS + 0.05% *v*/*v* Tween 20 + 1% *w*/*v* BSA, pH = 7.4, which flowed for 15 min at a flow rate of 30 μL/min. During this step, a signal was stabilized to establish the signal baseline.
2. The sample step: The sample was diluted at a ratio of 1:1 with PBS + 0.05% *v*/*v* Tween 20 + 1% *w*/*v* BSA, pH = 7.4. It flowed for 10 min at a flow rate of 30 μL/min. Binding of the analytes on the functionalized PIC surfaces occurred during this step.
3. The washing step: The buffer used was PBS + 0.05% *v*/*v* Tween 20 + 1% *w*/*v* BSA, pH = 7.4, which flowed for 15 min at a flow rate of 30 μL/min. Unbound viral particles and sample residues that could affect photonic measurements were removed at this step.
4. The PIC surface regeneration step: The buffer used was 50 mM Glycine + 50% *v*/*v* Ethylene Glycol, pH = 3, which flowed for 5 min at a flow rate of 30 μL/min. During this step, PIC surfaces were regenerated by releasing the captured antigens from the antibodies.
5. The final washing step: The buffer used was PBS + 0.05% *v*/*v* Tween 20, pH = 7.4, which flowed for 5 min at a flow rate of 30 μL/min. Removal of the regeneration buffer was critical for the preparation of PIC surfaces for the next experiments. In this step, BSA was excluded from the washing buffer to prevent protein accumulation in the microfluidics.

Outflows were delivered to a waste tank for UV sterilization. The total analysis time was 75 min, including the calibration of the device and the data analyses.

### 2.7. Data Fitting

The device’s detection system incorporates the LOWESS (Locally Weighted Scatterplot Smoothing) algorithm. LOWESS is used for the enhancement of the visual information of a scatterplot (such a scatterplot represents the data generated by the studied POC device) by computing and plotting smoothed points. Moreover, this algorithm smooths a scatterplot, (x_i_, y_i_), i = 1, …, n, in which the fitted value at x_k_ is a polynomial fit to the data using the weighted least squares method, where the weight for (x_i_, y_i_) is large if x_i_ is close to x_k_ and small if it is not. A robust fitting procedure is used to prevent the distortion of smooth points by deviant points [31]. For example, suppose that the input data has N points. The algorithm works by estimating the smooth y_i_ by taking the frac*N closest points to (x_i_, y_i_) based on their x_i_ values and estimating y_i_ by using a weighted linear regression. This indicates that the x_i_ values (in our case the minimum in nanometers) are not distorted by the algorithm. Therefore, the estimation of the shifts (estimated by the differences in x_i_ values of different analysis steps) is not affected by the implementation of the LOWESS algorithm.

### 2.8. Data Analysis and Shift Calculation

The data analysis was performed using a case-specific algorithm and novel software for PC, written in Python. This algorithm is also accessible through an android application and an online platform. To calculate the signal shifts in pm, photodiode responses in mV were plotted against their respective wavelength values in nm (laser sweeps in a wavelength range of approximately 1.5 nm). Minimum values of mV (notches) corresponded to specific wavelength values in nm. For both functionalized (R_functionalized_) and reference rings (R_reference_), the minimum wavelength values (in nm) were selected at two steps, the buffer step (Step 1—S1) and the washing step (Step 3—S3). Shifts of both functionalized and reference rings were calculated as the differences between the minimum wavelength values in steps 1 and 3 (D_functionalized_ and D_reference_, respectively). Relevant shifts caused by virus–antibody interactions were calculated by subtracting the absolute values of the two differences (|D_functionalized_|−|D_reference_|).

Positive relevant shifts corresponded to viral antigen detection, while negative relevant shifts corresponded to negative results. Relevant shifts were calculated for all functionalized rings, as rings operated independently.

### 2.9. Validation of the Device

To validate the device, 5 parameters were considered, namely, the Limit of Detection (LOD), sensitivity, specificity, accuracy, and precision.

#### 2.9.1. Limit of Detection—LOD

Cell culture supernatants for both PPV and PCV-2 were quantified using the previously described qPCR. To estimate the LOD, 6 serial 3-fold dilutions of cell culture supernatants in oral fluids were prepared, starting from 10^8^ viral copies/mL.

Afterwards, six PPV/PCV-2 functionalized PICs were tested against the 3-fold dilution panel. Positive shift results using the shift calculation equation were considered to indicate the presence of viral antigens.

#### 2.9.2. System Performance

The difficulty in fitting LOD data to a linear model led to the adoption of a qualitative system with a binary response variable (positive, negative) for the validation of the device. Consequently, the diagnostic performance of the device was assessed by estimating its sensitivity, specificity, accuracy, and precision. To estimate the numbers of True Positives (TP), True Negatives (TN), False Positives (FP), and False Negatives (FN), calibrators were classified in 3 categories based on the recommendations for the validation of diagnostic tests in clinical virology [32]. Positives (P) were considered samples that contained viral antigens at a dilution factor of more than 3 over the LOD of the test (PPV_LOD_ = 10^6^ copies/mL, PCV-2_LOD_ = 3.3 × 10^5^ copies/mL, see results). Low Positives (LP) were considered samples that contained viral antigens up to a dilution factor of 3 over the LOD of the test. Negatives were considered samples that tested negative with PCR methods. The sensitivity (TP/(TP + FN)), specificity (TN/(FP + TN)), accuracy ((TP + TN)/(TP + TN + FP + FN)), and precision TP/(TP + FP) were calculated and included both positive and low positive calibrators.

For the assessment of the system’s performance, 4 PICs were tested with a panel of spiked samples (Positive and Negative calibrators), and 2 PICs were tested with a panel of clinical samples (Low Positive calibrators) (Table 3). PICs could be used 6 times without compromising their performance. Although additional experiments could potentially have been performed, this was not attempted due to minor structural deterioration of the PICs. It is anticipated that having better manufacturing practices would increase the usable lifespan of PICs. Each PIC provided multiple independent measurements (based on the functionalized and reference ring combinations) for a single sample (see 2.8 Data analysis and shift calculation). The difference between the number of valid results obtained by PPV- and PCV-2-functionalized rings (n = 191 and n = 193, respectively) was due to functionalized rings that failed to produce normal signals.

### 2.10. Statistical Analysis

Mean shift values derived from LOD experiments were plotted against their respective viral concentrations (in log_10_ (Viral copies/mL)). The frequencies of test outcomes (TP, TN, FP, FN) were calculated to estimate the diagnostic performance of the device. Receiver Operating Characteristic (ROC) curves were calculated to estimate the area under the curve (AUC) and the respective 95% Confidence Intervals (95% CI) for both PPV and PCV-2. The statistical analysis was performed with SPSS v23 software (IBM Corp., Armonk, NY, USA).

## 3. Results

### 3.1. PCR Results

**Figure 4:**
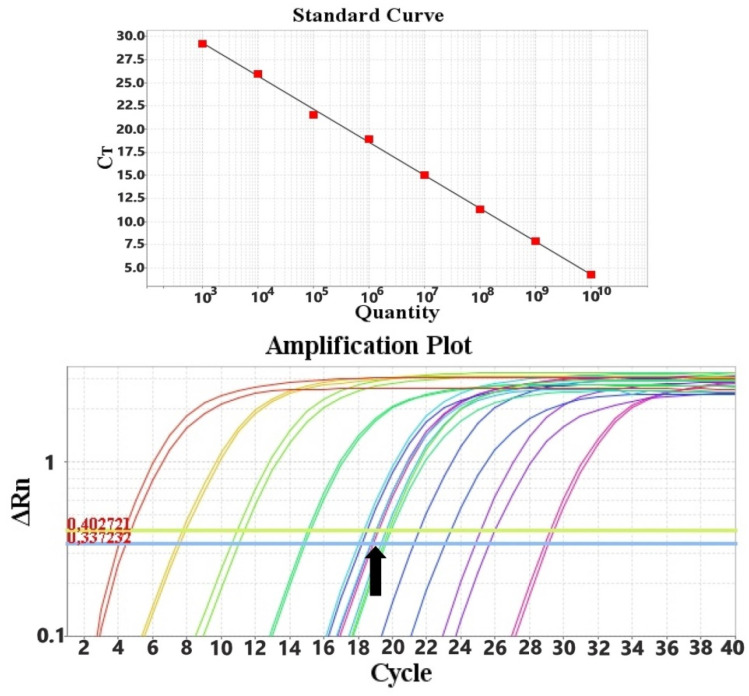
qPCR standard curve using the PPV_Set_1 primer set, which targets the NS1 gene, and the amplification plot with reference and field samples indicated with the black arrow.

**Figure 5:**
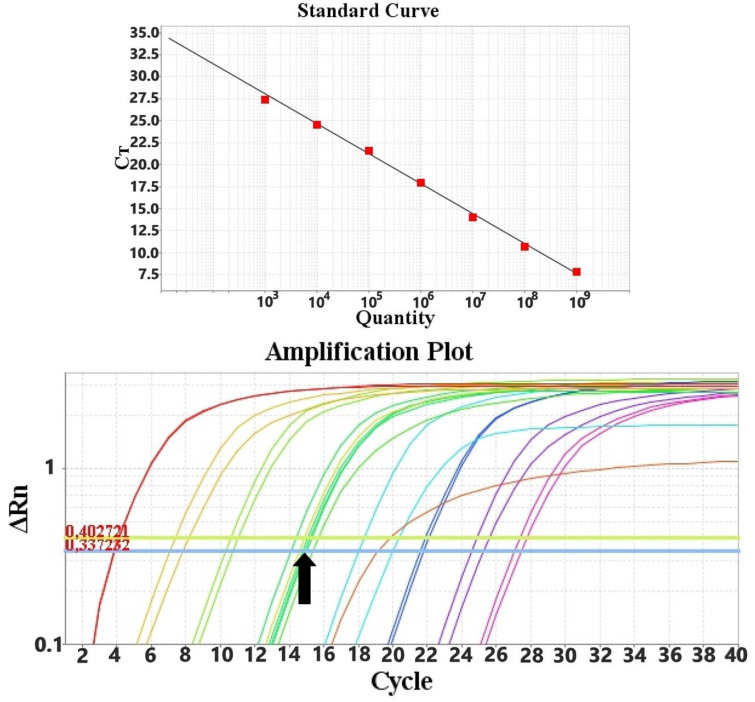
qPCR standard curve using PCV-2_Set_1 primer set, which targets the capsid protein gene, and amplification plot with reference and field samples indicated with the black arrow.

Out of the 155 collected samples, 75 were oral fluid and 80 were fecal samples. All samples were screened with conventional PCR for both PPV and PCV-2. PPV was detected in 7/75 oral fluid samples (9.3%) and 8/80 fecal samples (10.0%). PCV-2 was detected in 15/75 oral fluid samples (33.3%) and 27/80 fecal samples (33.7%). PPV and PCV-2 co-infection was observed in 4/75 (5.3%) oral fluid samples and in 7/80 (8.7%) fecal samples. All positive PPV samples were collected from 1 farm and PCV-2 positive samples were collected from 2 farms, out of the 6 farms included in the study. Positive samples were quantified with the SYBR Green-based qPCR, using the following standard curves produced in this study (Figure 4 and Figure 5).

### 3.2. Data Fitting

**Figure 6:**
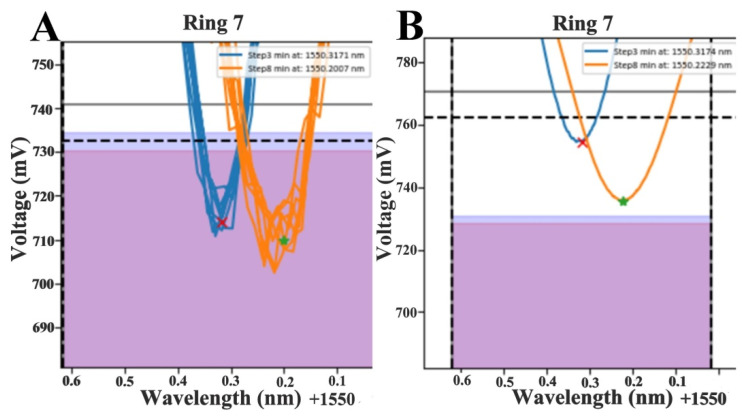
Application of the LOWESS algorithm to raw data: (**A**) data prior to algorithm implementation, (**B**) data after the implementation of LOWESS. The wavelength values (x-axis) used for shift estimation remained identical.

The results of the application of the LOWESS algorithm to raw data (real measurements with functionalized rings) for optimization of the visual interpretation of results and accurate shift estimation are presented in the following image (Figure 6).

The minimum values selected after the implementation of the algorithm were closer to the mean value of repeated measurements. The variation in the x-axis (wavelength used for the estimation of shifts) was minimal (almost non-existent), as the algorithm weighs the data based on their x_i_ values. On the contrary, there was observable variation in the y axis values. However, the variation of the y axis values did not affect the estimation of the shifts.

### 3.3. Limit of Detection-LOD

**Figure 7:**
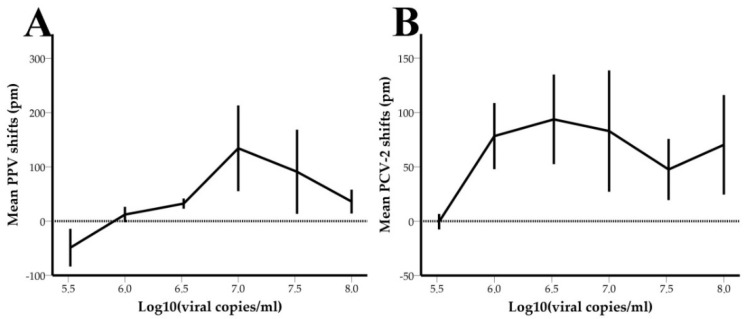
PPV (**A**) and PCV2 (**B**) shift responses (in pm) plotted against viral concentrations (log_10_ (Viral copies/mL)).

In the images below (Figure 7), the shift responses (in pm) of PPV- and PCV-2- functionalized sensors are plotted against the viral concentrations (expressed in log_10_ (Viral copies/mL)) of oral fluid samples. The error bars represent the standard errors of the shifts for each viral concentration.

PPV and PCV-2 functionalized rings showed a LOD of 10^6^ copies/mL and 3.3 × 10^5^ copies/mL, respectively, which was determined as the lowest viral copy number that produced positive shift values.

### 3.4. Performance of the System

To estimate the performance of the device, TP, TN, FP, and FN were calculated by comparing the shift responses of samples to the detection thresholds for PPV (4.5 pm) and PCV-2 (6.5 pm) (see Section 3.5, ROC curves). The screening results obtained using the POC device are summarized in Table 4 and Table 5. The performance metrics of the device for PPV and PCV-2 are summarized in Table 6. Additionally, 10 PPV-positive and 10 PPV-negative fecal samples were tested, while 16 fecal samples positive for PCV-2 were tested with the device. Out of the ten (n = 10) PPV positive samples, seven (n = 7) gave a true positive result and three (n = 3) were false negatives, while out of the ten (n = 10) PPV negative samples, nine (n = 9) gave a true negative result and one (n = 1) gave a false positive result. Nine of the PCV-2 positive fecal samples (n = 9) gave a true positive result and seven (n = 7) gave a false negative result.

### 3.5. Receiver Operating Characteristic (ROC) Curves

**Figure 8:**
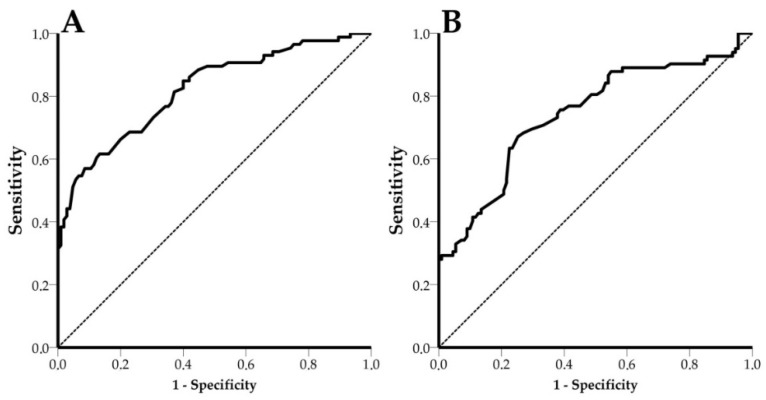
ROC curve for PPV and PCV-2. The dashed line represents the diagonal reference line. (**A**) PPV ROC curve, AUC = 0.820, CI: 0.760 to 0.880, *p* < 0.0001 and (**B**) PCV-2 ROC curve, AUC = 0.742, CI: 0.670 to 0.815, *p* < 0.0001.

For estimation of the AUC, 191 and 193 shift values for PPV and PCV-2, respectively, were used. In the case of PPV, an AUC value of 0.820 (95% CI: 0.760 to 0.880, *p* < 0.0001) and an optimal shift efficiency threshold equal to 4.5 pm (68.6% sensitivity, 77.1% specificity) were estimated (Figure 8). PCV-2 had an AUC value of 0.742 (CI: 0.670 to 0.815, *p* < 0.0001) and an optimal shift efficiency threshold equal to 6.5 pm (69.5% sensitivity and 70.3% specificity) (Figure 8). In both cases, optimal thresholds were considered those with shifts greater than zero, in order to produce meaningful results.

**Figure 9:**
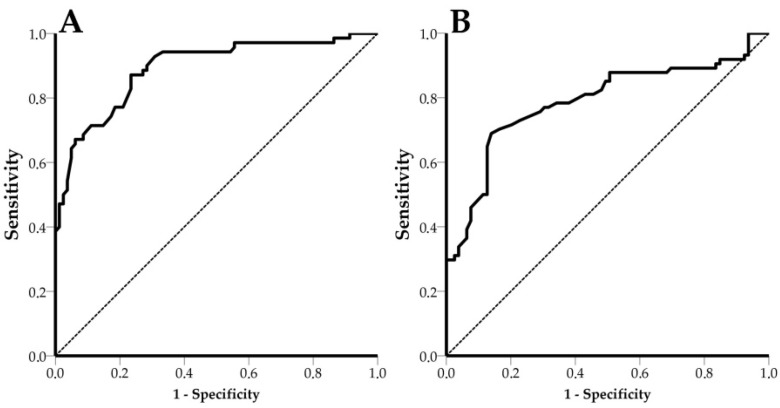
ROC curves for PPV and PCV-2 after the exclusion of the outlier, PIC #45. The dashed line represents the diagonal reference line. (**A**) PPV ROC curve, AUC = 0.892, CI: 0.840 to 0.944, *p* < 0.0001 and (**B**) PCV-2 ROC curve, AUC = 0.788, CI: 0.712 to 0.863, *p* < 0.0001.

Among the 6 PPV/PCV-2 functionalized PICs, PIC #45 showed extremely poor performance, affecting AUC values for both PPV and PCV-2. Excluding PIC #45 from the ROC analysis as an outlier (and, consequently, from the system’s diagnostic performance assessment) significantly improved the results. In detail, PPV had an AUC value of 0.892 (95% CI: 0.840 to 0.944, *p* < 0.0001) and an optimal shift efficiency threshold equal to 4.5 pm (77.1% sensitivity, 81.5% specificity) (Figure 9). PCV-2 had an AUC value of 0.788 (CI: 0.712 to 0.863, *p* < 0.0001) and an optimal shift efficiency threshold equal to 6.5 pm (71.6% sensitivity and 79.7% specificity) (Figure 9).

## 4. Discussion

The present study demonstrated that PICs, and photonics in general, can be combined with emerging technologies (microfabrication, microfluidics, modern data acquisition, and communication strategies) to be successfully integrated into a compact, portable device that is fit for POC testing, for the label-free detection of swine viral diseases in oral fluids. The diagnostic performance of the device was satisfying, achieving sensitivity values of 68.6% and 69.5%, specificity values of 77.1% and 70.3%, and AUC values of 0.820 and 0.742 for PPV and PCV-2, respectively. Further testing and optimization of PIC and device fabrication procedures are expected to significantly improve the performance of the device.

The control of swine viral diseases is heavily dependent on early and reliable diagnosis, which is crucial for the mitigation of transmission risk within the swine population [33]. POC testing is envisioned to meet this demand with the development of cost-effective tests performed under farm conditions. However, most POC tests for animal diseases suffer from limitations, such as low sensitivity, cost, complexity, extended analytical time, a limited number of targeted analytes, a lack of field testing, and improper validation. In many cases, this results in low-quality tests entering the market [34]. The need for next-generation POC devices with extended capability for the detection of swine viral diseases is reflected by the willingness of consumers to invest up to 5000 euros in similar equipment [35]. 

To tackle these limitations, the novel system utilizes modern microfabrication technologies. Microfabrication allows the production of sensors at the μm or even the nm scale, which can detect interactions of biomolecules and transduce them into a measurable signal. PICs fabricated with this technology were found to be ultra-sensitive to changes in the refractive index, providing a robust platform for the detection of interactions between the antibodies immobilized on PIC surfaces and viral antigens.

Microfluidics and information and communication technologies were used in our studies to completely automate the analysis (delivery of fluids, measurements, and data analysis), allowing operation via an android-launched application. End-users need to collect oral fluid samples using cotton ropes, dilute the samples to a 1:1 ratio with PBS + 0.05% *v*/*v* Tween 20 + 1% *w*/*v* BSA at pH = 7.4, and filter them using 5 μm and 0.45 μm pore size syringe filters, in sequence. Afterwards, 0.6 mL of the diluted and filtered sample is placed in an Eppendorf tube and inserted into a sample holder. Two more Eppendorf tubes, containing 1.2 mL of the analysis buffer and 0.6 mL of the regeneration buffer are placed in their respective positions to a buffer holder. Finally, 1 mL pipette tips are inserted into a tip holder. The sensors are connected by hand to the optic fibers and the microfluidic system with simple male/female connectors and then covered with insulation foam to maintain a constant temperature of 25 °C (analysis temperature). After completing this handling, the device is operated via a tablet, making it operator friendly. The device provides a simple positive or negative result, diminishing user-introduced biases in the applied analytical procedures and the interpretation of results. Therefore, the device can be used by nonspecialized personnel without impacting its performance remarkably, which is not always the case in POC diagnostics.

To facilitate and simplify data analysis, we exploited the LOWESS algorithm, which calculates shifts in curves of continuously measured refractive indexes. The algorithm also aids in the interpretation of results, improving automation and the efficiency of the device. Results generated by the device are stored online using a cloud platform and appropriate data transferring applications. Cloud storage facilitates the meta-analysis of results for the establishment of effective surveillance protocols against targeted diseases and allows further development of telemedicine. The system has a modular approach, allowing easy servicing and replacement of broken parts. The integrated syringe-based, fluid delivery system is inexpensive compared to other solutions used to deliver fluids, such as peristaltic pumps. The overall architecture and design of the system enables the manufactured device to be deployed directly on farms, peripheral and mobile laboratories, and border checkpoints for the sensitive and fast detection of swine viral diseases. These features make the system one of the first next-generation POC devices to be introduced to veterinary diagnostics.

The novel POC device was validated using PPV and PCV-2 in complex biological samples as proofs of concept. PPV is a small, nonenveloped, negative ssDNA virus that is associated with reproductive failure in swine and is the causative agent of SMEDI syndrome [36]. PPV is an extremely stable virus in pig slurry, staying infective for more than 40 weeks at 20 °C and requiring exposure to 50–55 °C for a week to be completely inactivated [37]. Moreover, strains traditionally used in vaccines seem to be ineffective against newly-emerged, highly virulent strains [38]. Therefore, early diagnosis and screening could play a major part in controlling the disease. In this study, PPV was detected in oral fluids, confirming the findings of Milek et al. [39].

PCV-2 is a small, nonenveloped virus with a circular, single-stranded DNA genome. PCV-2 has been associated with postweaning multisystemic wasting syndrome (PMWS), porcine dermatitis and nephropathy syndrome (PDNS), and reproductive disorders [40]. Vaccines have been able to improve the daily weight gain and prevent the manifestation of clinical disease. Nonetheless, repeated exposure to the pathogen and other cofactors may render vaccines ineffective in practice [41]. 

Isolated PPV and PCV-2 infections are not associated with severe clinical disease and high mortality rates [9,10]. However, both viruses can place a great burden on the financial sustainability of swine farms, mainly by decreasing weight gain. In certain cases, high levels of viral copies in oral fluids (>10^6^ copies/mL) for PPV and PCV-2 can cause intense clinical symptoms [42,43,44]. The viral copy number induced by the clinical manifestation of these diseases coincides with the LOD of the system. Quantification of results was not possible for the selected range of viral concentrations due to the prozone effect, as visualized by the characteristic hook-shaped curve in the LOD experiments. The prozone effect generates similar shift responses when samples of different viral copy numbers are tested. This phenomenon could be bypassed by increasing the dilution factor of samples to reduce the viral concentration.

The device had a satisfactory performance when tested with spiked and clinical (low calibrators) oral fluid samples. Although the performance metrics may seem suboptimal, it is important to highlight the framework in which these results were generated. PCR, an extremely sensitive method, was used as the “gold standard”, resulting in lower sensitivity and specificity levels for the device, as expected. PCR is currently the most commonly used method for PPV and PCV-2 detection and quantification in veterinary practice, effectively making PCR the only “gold standard” that would produce meaningful information for end-users.

The assessment of the performance of the system should be based on its intended use as a field-based POC device and the innovations that it can introduce into the field of animal diagnostics. To our knowledge, this is the first attempt to sense pathogens affecting animals in complex biological samples using PICs in the POC setting. PPV can be detected in oral fluids and feces; however, further testing is required with fecal samples. PCV-2 can only be detected in oral fluids. Oral fluids and feces have been proven to be useful alternatives to sera and tissues for conventional (e.g., PCR) and photonic methods of detection, as both sample types are not intrusive, are easy to collect, are suitable for herd screening, and are cost-effective [45,46,47].

Furthermore, it is important to note that the aforementioned sensitivity and specificity levels were derived when PIC #45 was included (PPV_PIC#45_: 5 TP, 15 TN, 9 FP, 11 FN, PCV-2_PIC#45_: 4 TP, 15 TN, 17 FP, 4 FN). The ROC curve analysis showed that the AUC, sensitivity, and specificity values were significantly improved when PIC #45 was excluded, underlining the importance of PIC manufacture. PIC homogeneity and manufacturing methods play major role in photonic sensing, considering that results are generated at the scale of nanometers. Increasing the Technology Readiness Level (TRL) of the device (current TRL = 6) would help to standardize and automate the PIC manufacturing chain [48] and further improve the sensing platform.

Further tests to improve the device and the PICs and to allow the semi-quantification of results are underway. Automated fabrication of PICs and standardization of materials/procedures could further reduce manufacturing tolerance. Sensitivity, specificity, and LOD levels of sensors could be improved by immobilizing antibodies on ring resonators in an oriented way. The immobilization of antibodies and blocking proteins (reference rings) with 3D microprinters is expected to refine signal resolution and reduce the background. Lastly, the selection and testing of new molecular recognition elements (MREs), that recognize different antigen epitopes or strains could be deployed on functionalized rings, significantly improving the performance of the sensors. 

Currently, the device has been developed for the detection of six major swine viral pathogens, namely Porcine Parvovirus (PPV), Porcine Circovirus 2 (PCV-2), Classical Swine Fever Virus (CSFV), Porcine Respiratory and Reproductive Syndrome (PRRSV), Swine Influenza Virus (SIV), and African Swine Fever Virus (ASFV). PICs can be functionalized for two or more diseases (PPV, PCV-2, SIV and ASFV use compatible buffers in analysis), and the device can test up to 4 PICs simultaneously, allowing the multiplex detection of viral pathogens. PICs could be functionalized to target other analytes such as antibodies and/or different antigens, extending its utility beyond the limits of swine farm biosecurity. Alternative samples, such as sera, swabs, and feces, could be used with adaptations in the analysis protocol, tailoring sampling protocols to the respective animal species. These adaptations include the use of alternative buffers, an increase in sample dilution factors, and sample pre-treatment.

Overall, the novel POC system is a promising method for the sensitive and specific detection of viral pathogens. This system can reduce screening costs and minimize the time required for the diagnosis of viral diseases. Specifically, for PPV and PCV-2, the device can be utilized at the farm level to evaluate the health status of newly purchased animals and to identify PPV- and PCV-2-infected animals before the onset of clinical disease, to support evidence-based disease control strategies. 

Future research should focus on the production and characterization of MREs to widen the panel of detectable diseases, the optimization of small-scale manufacturing methods, and the orientation of antibodies. Finally, research should be done to increase the number of tests and sample types to allow the investigation of system/sensor limitations that were not detected in this study.

## 5. Conclusions

It is expected that emerging sensing and manufacturing technologies will be integrated into compact devices for reliable and cost-effective POC testing. User-centered devices and tests offer veterinarians and farmers the opportunity to perform rapid, on-field diagnoses and, consequently, to implement evidence-based prevention and control protocols. In this context, the system paves the way for next-generation POC testing by bringing veterinary diagnostics to the crossroads of biology, mechanics, and photonics. The current limitations of the system are expected to be improved with future investments and research, which will increase the TRL of the device. Novel POC technology offers a portable system for the photonic detection of various animal pathogens, extending beyond the limits of swine-relevant diseases by altering the functionalization of PICs and further contributing to the optimization of livestock biosecurity.

## Figures and Tables

**Table 1:** Primer sets used in conventional PCR for PPV and PCV-2 detection.

Primer Set	Target	Primer Sequence (5′-3′)	Amplicon Length (bp)	Literature
PPV_Set_1	NS1 gene	Forward: TTGGTAATGTTGGTTGCTACAATGC Reverse: ACCTGAACATATGGCTTTGAATTGG	127	[24]
PPV_Set_2	NS1 gene	Forward: AGCCAAAAATGCAAACCCCAATA Reverse: CTCCACGGCTCCAAGGCTAAAG	142	[25]
PPV_Set_3	NS1 gene	Forward: ATACAATTCTATTTCATGGGCCAGC Reverse: TATGTTCTGGTCTTTCCTCGCATC	330	[24]
PCV2_Set_1	PCV-2 Capsid protein gene	Forward: TAGGTTAGGGCTGTGGCCTT Reverse: CCGCACCTTCGGATATACTG	263	[26]
PCV2_Set_2	PCV-2 Rep gene	Forward: CACATCGAGAAAGCGAAAGGAAC Reverse: TGCGGGCCAAAAAAGGTACAGTT	505	[27]
PCV2_Set_3	PCV-2 ORF1	Forward: GCCAGTTCGTCACCCTTTC Reverse: CTCCCGCACCTTCGGATAT	657	[28]

**Table 2:** Cycling conditions for each primer set.

Primer Set	Pre-Denaturation at 95 °C	Cycles	Denaturation at 94 °C	Annealing for 30 s at	Extension at 72 °C	Final Extension at 72 °C
PPV_Set_1	2 min	32	20 s	62 °C	30 s	1 min
PPV_Set_2	2 min	32	20 s	59 °C	30 s	1 min
PPV_Set_3	2 min	32	20 s	62 °C	30 s	1 min
PCV2_Set_1	2 min	32	20 s	60 °C	30 s	1 min
PCV2_Set_2	2 min	32	20 s	62 °C	40 s	1 min
PCV2_Set_3	2 min	32	20 s	59 °C	40 s	1 min

**Table 3:** Calibrators included in the calculation of TP, TN, FP, and FN.

	Positive and Negative Calibrators			Low Positive Calibrators		
Exp. Number	Sample Type	Negative/Positive	Viral Copies/mL	Sample Matrix	Virus	Viral Copies/mL
1st	Oral fluids	Negative for PCV2 & PPV	0	Oral fluids	PPV, PCV-2	7 × 10^5^ 9 × 10^5^
2nd	Oral fluids	Positive for PCV2 & PPV	10^8^	Oral fluids	PPV, PCV-2	9 × 10^5^ 3 × 10^6^
3rd	Oral fluids	Negative for PCV2 & PPV	0	Oral fluids	PPV, PCV-2	1.1 × 10^6^ 4 × 10^6^
4th	Oral fluids	Positive for PPV	10^8^	Feces	PCV-2	6 × 10^6^
5th	Oral fluids	Negative for PCV2 & PPV	0	Feces	PPV, PCV-2	9 × 10^5^ 4 × 10^6^
6th	Oral fluids	Positive for PCV2	10^8^			

**Table 4:** Results of the performance assessment for PPV using the POC system.

		Status of Samples According to PCR–PPV		
		Positives	Negatives	Total
Screening results	Positives	59 (TP)	24 (FP)	83
	Negatives	27 (FN)	81 (TN)	108
	Total	86	105	191

**Table 5:** Results of the performance assessment for PCV-2 using the POC system.

		Status of Samples According to PCR–PCV-2		
		Positives	Negatives	Total
Screening results	Positives	57 (TP)	33 (FP)	90
	Negatives	25 (FN)	78 (TN)	103
	Total	82	111	193

**Table 6:** Performance metrics summary of the novel POC device regarding the detection of porcine parcovirus and porcine circovirus 2.

Metrics	PPV %	PCV-2 %
Sensitivity	68.6	69.5
Specificity	77.1	70.3
Accuracy	73.3	69.9
Precision	71.1	63.3
